# Elastic, not plastic species: Frozen plasticity theory and the origin of adaptive evolution in sexually reproducing organisms

**DOI:** 10.1186/1745-6150-5-2

**Published:** 2010-01-13

**Authors:** Jaroslav Flegr

**Affiliations:** 1 Department of Philosophy and History of Science, Charles University, Viničná 7, CZ-128 44 Praha 2, Czech Republic

## Abstract

**Background:**

Darwin's evolutionary theory could easily explain the evolution of adaptive traits (organs and behavioral patterns) in asexual but not in sexual organisms. Two models, the selfish gene theory and frozen plasticity theory were suggested to explain evolution of adaptive traits in sexual organisms in past 30 years.

**Results:**

The frozen plasticity theory suggests that sexual species can evolve new adaptations only when their members are genetically uniform, i.e. only after a portion of the population of the original species had split off, balanced on the edge of extinction for several generations, and then undergone rapid expansion. After a short period of time, estimated on the basis of paleontological data to correspond to 1-2% of the duration of the species, polymorphism accumulates in the gene pool due to frequency-dependent selection; and thus, in each generation, new mutations occur in the presence of different alleles and therefore change their selection coefficients from generation to generation. The species ceases to behave in an evolutionarily plastic manner and becomes evolutionarily elastic on a microevolutionary time-scale and evolutionarily frozen on a macroevolutionary time-scale. It then exists in this state until such changes accumulate in the environment that the species becomes extinct.

**Conclusion:**

Frozen plasticity theory, which includes the Darwinian model of evolution as a special case - the evolution of species in a plastic state, not only offers plenty of new predictions to be tested, but also provides explanations for a much broader spectrum of known biological phenomena than classic evolutionary theories.

**Reviewers:**

This article was reviewed by Rob Knight, Fyodor Kondrashov and Massimo Di Giulio (nominated by David H. Ardell).

## Background

### Problems of Current Models of Adaptive Evolution in Sexual Species

Darwin's evolutionary theory[1] could easily explain the evolution of adaptive traits (organs and behavioral patterns) in asexual organisms. However, its capacity to explain the origin of such traits in sexual organisms, i.e. in most eukaryotic species on Earth, was already widely discussed by Darwin's critics in the 19^th ^century[2]. The most frequent objection was that in sexual organisms phenotypes, as well as forms of particular traits, have a continuous tendency to return to original state, as the carriers of a new phenotype (a new trait) mate with more numerous carriers of the old phenotype. It is possible to show that such "averaging" of phenotypes by sexual reproduction would result in the loss of one half of the contemporary phenotypic variability in each generation[3]. This objection died away after the rediscovery of Mendel's genetic laws. The corpuscular nature of the gene was generally considered to be an adequate solution to the problem of the blending inheritance of biological traits[4]. Mendelian genetics showed that despite a, sometimes intermediate, phenotype of crossings, the genes responsible for a particular form of trait from two parents do not interact in any way in bodies of crossings and are transmitted to the next generations in their original form. If a new form of a gene originated by a random mutation event encodes a useful character, it improves the viability or fertility of the organism and is therefore transmitted to the next generations in more copies than the original form of the gene. As late as the mid 1970s, Richard Dawkins clearly and loudly enough claimed that the solution provided by Mendelian genetic is just illusory[5]. It could solve the problem of vanishing genetic variability; however, it could not solve the problem of vanishing inheritability of large parts of phenotypic traits and therefore vanishing inheritance of fitness. Darwin's model of evolution of adaptive traits based on intra-population competition of individuals for highest fitness expects that fitness is inherited from parents to offspring. If an individual has an allele that gives it an extraordinary fitness (above-average viability or fertility), it not only transmits more copies of this allele to the next generation, but also its offspring have above-average fitness and transfer this allele to the next generations in an above average number of copies. Due to this mechanism, the number of alleles coding for useful (adaptive) traits should continuously grow from generation to generation. The problem is that in sexually reproducing organisms neither genotype nor phenotype is inherited from parents to offspring, and even less is fitness. The genotype of any offspring is always unique as it is always generated *de novo *by random combinations of genes from the two parents. This phenomenon has a very important effect, namely, even an excellent individual showing extremely high fitness may have offspring whose fitness is average or even subnormal. Usually, within a few generations the unique combination of genes of an excellent individual will be diluted and its relatives will in no way differ from relatives of any other individual.

Dawkins not only identified a weakness of the traditional individual competition-based evolutionary model, but also suggested a new model of the evolution of adaptive traits based on intralocus competition of alleles for the highest number of copies transferred to the next generation - the selfish gene theory[5]. Dawkins argued that in contrast to genotype, which is not inherited but originates *de novo *in each generation by mixing alleles from two parents, an allele is nearly always transmitted to offspring in an unchanged form. Therefore, when a new allele increases the efficiency of transfer to the next generation by, for example, increasing the viability of its carrier, it will spread in the gene pool of a population by intralocus competition. The theory of intralocus competition (or selfish gene), which was implicitly used before Dawkins by G.C. Williams[6] and W.D. Hamilton[7,8], is better than the original theory of intrapopulation individual selection as it offers a unified theoretical framework for explaining a broader spectrum of evolutionary phenomena. It explains not only the evolution of adaptive traits but also the evolution and spread of certain alleles which decrease the fitness of their carrier (outlaw genes in Dawkins's terminology) as well as the spread of a category of altruistic genes, i.e. alleles for behavior that decrease the direct fitness of their carrier while increasing its inclusive fitness by increasing the direct fitness of its relatives. The selfish gene theory is now mainstream evolutionary theory. *When a classical evolutionary biologist looked for the purpose of a trait, his/her question was how does this trait enhance an individual's fitness. When a current evolutionary biologist is looking for the purpose of such a trait, his/her question is how does this trait increase the number of copies of the gene variant involved in the formation of this trait*.

Dawkins supposed that the model of intralocus selection is also a solution to the problem of vanishing inheritance of fitness in sexual organisms. However, this opinion is most probably wrong. It is true that, in contrast to genotype, an allele is nearly always transmitted from parents to offspring in an unchanged form. *Three important reasons exist why the same allele has a different influence on fitness in parents and in offspring. These are: (1) dependence of the fitness of carriers of a particular allele on the frequency of this allele in the population (2) dependence of the phenotypic expression of an allele on the genotype of the individual (3) dependence of the effect of a trait on fitness on the phenotype of the individual*.

Population genetics models usually suggest that each allele can be characterized by a constant, a value that describes the average relative fitness of the carriers of a particular allele. This common approach, however, oversimplifies the situation in real systems. In such systems, some decreasing or increasing function of the frequency of an allele, rather than a constant, usually describes the influence of an allele on the fitness of its carriers[9,10], i.e. the so called frequency dependent selection is operative, rather than regular directive selection [9,10]. For example, when the frequency of the *s *allele for sickle cell disease is low in a population living in an endemic malaria area, the allele has a highly positive value for its carriers[11]. Sexual partners of carriers of this allele are homozygotes with two normal alleles; and therefore, only heterozygotes with higher tolerance to malaria, and not homozygotes with two *s *alleles and therefore with the fatal form of sickle cells disease, will occur among their offspring. When the frequency of the *s *allele increases, it losses its positive value for carriers as many homozygotes, with the fatal form of sickle cell disease, will spring out among the offspring of a heterozygote.

Similarly, an allele for the exploitation of an alternative resource is very useful when rare; however, it can be much less useful or can even be harmful when it turns common, because too many carriers of this allele can use up the resource. As shown by Price and Maynard Smith[12,13], in the case that a biological value of the strategy (the trait) depends on the frequency of this, and of other strategies in the population, the evolutionarily stable strategy wins the competition in the long run. In the case of genetically coded strategies, the winners of the evolutionary game would be such alleles that, when they prevail in the population, cannot be invaded by any alternative strategy that is initially rare. It is clear now that this kind of stability, rather than traditional fitness, is the most important criterion of evolutionary success in the competition between gene variants.

Price and Maynard Smith described the competition of strategies using the hawk and dove model. We have a population of hawks and doves that represent two distinct behavioral strategies in combat. When two doves find a piece of food, they peacefully share it and each of them receives, on average, one half the benefit. When a dove and a hawk find a piece of food, the hawk drives away the dove to get all the benefit for itself. When two hawks find a piece of food, they fight for it; and each of them receives, on average, one half the benefit and pays one half the cost - the cost here means time lost as well as possible injuries sustained. It is a major asset to be a hawk in a population of doves because such a hawk will prevail in all combat without cost. At the same time, it is an asset to be a dove in a population of hawks, especially when the cost of combat is very high, because the dove never pays the cost of combat while hawks have to pay this cost in nearly all combat. From this it follows that neither the dove nor the hawk strategy can win the competition and some equilibrium frequency of the strategies will be established. When mixed strategies are allowed, then the so-called evolutionarily stable strategy wins. Here the evolutionarily stable strategy means "behave with frequency benefit/cost as a hawk and with frequency 1 - benefit/cost as a dove".

Because of the nature of the original model of Price and Maynard Smith, and also because most people conceive of strategy only in the context of intentional behavior, the area of evolutionarily stable strategies is mostly perceived as part of behavioral biology. However, in fact, the same laws rule the evolution of any trait whose biological value (selection coefficient) depends on its frequency in the population[14], including evolution of many ontogenetical traits[15]. It is highly probable that this is true of many traits in asexual organisms and of most traits in sexual organisms. It is beneficial to be a carrier of a rare strategy in an asexual organism, as this enables the carrier to exploit underexploited resources as well as to avoid enemies whose combat strategies are specialized to exploit a feature of the common form of the organism. In sexually reproducing organisms, the competition between strategies for this kind of stability probably controls the evolution of all polymorphic genes, i.e. of most of genes[16]. In the previous model, the hawks and doves meet each other over a piece of food. In sexual species, pairs of alleles of different genes meet after male and female sex cells combine in newly formed zygotes. The competition between alleles therefore corresponds to that between hawks and doves shown in the model. For each allele, an equilibrium frequency exists that can be shifted, for example by selection; however, it will be restored after the end of the selection.

At face value, there is an important difference between the situation in the hawk and dove game and the sperm and oocyte game. If a dove and a hawk meet over a piece of food, each takes away a different reward from the conflict. On the other hand, if an individual is a heterozygote, i.e. if normal alleles and *s *alleles meet in his cells, it might seem that both alleles take the same reward from the meeting, as his fitness affects the evolutionary fate of both alleles to the same degree. In actual fact, this need not be true. Imagine, for example, that a large percentage of the zygotes carrying two *s *alleles do not settle in the sex organs of the woman and die without substantially utilizing the resources of the maternal organism (if we neglect the fact that the woman does not become pregnant in that month). If embryos with two *s *alleles are rarely implanted in the uterus or embryos with two *s *alleles are frequently aborted, then the consequences to each of the two alleles in the meeting of normal and *s *alleles in the genome of a heterozygote woman, will differ. If the partner of the woman is also a heterozygote, then the number of descendants carrying a copy of the normal maternal alleles will be greater than that of descendants carrying copies of her *s *alleles. This is because the embryos with the maternal *s *alleles, which also carry the paternal *s *alleles, will most probably be aborted. However, if the partner is a homozygote with two normal alleles, the mother will transfer to her descendants the same number of copies of both her alleles. On the other hand, such descendants carrying copies of normal alleles will frequently die of malaria in childhood, while the descendants carrying copies of the *s *alleles will be far more likely to live to a reproductive age. The rules of the relevant evolutionary game are thus, in actual fact, far more complicated than in the dove and hawk model. Additionally, we are, for the present, completely ignoring the fact that both the alleles of a single gene as well as the alleles of various genes can interact in their effects. Thus, the fitness of the carriers of certain alleles very frequently depends on which alleles are present in the other parts of the genome. Because of pleiotropy, the effects of a gene on many different traits, it is highly probable that the frequencies of most alleles in sexual species are kept at a certain value by a complex network of competing or supporting strategies. If a new mutation with a frequency-independent effect on fitness occurs in a population, it is either eliminated or fixed by selection or by drift. If a new mutation with frequency-dependent effect occurs in a population, it most probably snarls up in the existing network of frequency-dependent strategies. In large populations, where selection rather than drift governs the destiny of most alleles, this mechanism could be responsible for the maintenance of most alleles in a polymorphic state.

**Figure 1:**
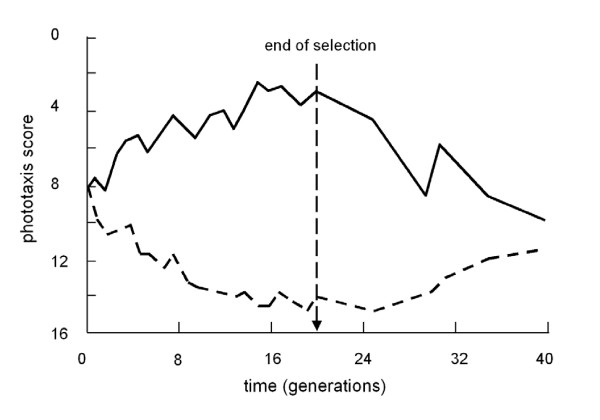
**Elastic response of laboratory populations on artificial selection for positive and negative phototaxis**. A population of *Drosophila pseudoobscura *was divided into two parts, one of which was subjected to long-term selection for positive phototaxis - movement towards light in a Y-maze (broken line) and the second to negative phototaxis - movement into darkness (solid line). During twenty generations, the population diverged substantially in this trait. After termination of selection, the average phenotypes in both populations returned to the original value during the subsequent twenty generations. Data from Dobzhansky and Spassky[17].

The effect of a frequency-dependent selection on the response of a population to selection can be observed in usual selection experiments[17] (Fig. 1). In such experiments we can, for example, select large drosophilas by killing all small flies. This is analogical to shooting the hawks in Price-Maynard Smith's model population. The pay-off matrix in the game is consequently changed; to be a hawk, as well as to be an allele for small body size, will become less advantageous than it was during the equilibrium that existed before the selection started. The frequency of such allele and therefore also the average body size of our drosophilas decreases. However, as the frequency of this allele goes down, the advantage of being such allele (of being a hawk) goes up. Therefore, our selection is decreasingly less effective and, at one point, our population stops responding to the selection. When we terminate the experiment, when we stop killing small drosophilas or stop shooting hawks, the frequency of the given allele (of hawks) will return to the equilibrium value. This means that the population of sexual organisms under artificial or natural selection mostly behaves elastically rather than plastically. The existence of a so-called genetic homeostasis, as well as a post selection return to the original state, was described in the middle of the last century[18]; likewise, the resistivity of many populations (with polymorphism in a selected trait and high heritability of the trait) to artificial or natural selection is a common problem of current evolutionary biology, for review see[19] and breeding practice [20-22].

Frequency-dependent selection is just one of three complications of the solution to the problem of the vanishing inheritance of fitness suggested by the selfish gene theory. Another complication, widely discussed by evolutionary and theoretical biologists for the past 50 years, is gene interactions. The relationship between genes and traits is not as straightforward as is supposed by most evolutionary models, including the selfish gene theory (see also [23,24]). Due to pleiotropy, one gene usually influences a number, sometimes even a large number, of traits and due to epistasis, one trait is usually influenced by multiple genes. Moreover, the influences of particular genes on a trait are often not additive. The same allele in the context of one combination of alleles (of the same or of other genes) influences a given trait, for example body size, positively; while in the context of another combination of alleles it may influence the same trait negatively[25]. Consequently, there is no such thing as an allele, or a mutation, for large body size. The phenotypic expression of most alleles is in fact genotype-conditioned. In the normal natural population in which a great quantity of genetic polymorphism is sustained by means of different forms of frequency-dependent selection, the same allele probably changes not only the value but also often the sign of its effect on the corresponding trait from generation to generation as it "travels" from genotype to genotype.

Last, but possibly not least, the effect of the same trait on the fitness of an individual is usually dependent on combination of other traits borne by the same individual[26]. In the context of certain combination of traits (for example in the context of high physical strength) a particular trait (for example aggressiveness) is useful while in the context of other traits (in a weak individual) the same trait (aggressiveness) could dramatically decrease fitness of an individual. Dawkins was right in his claim that in contrast to genotype, an allele is transmitted from parents to offspring unchanged even in sexual organisms. However, in genetically polymorphic species, i.e. in normal sexually reproducing species, the effect of the same allele on phenotype, or at least on fitness, varies from generation to generation. This makes the gradual evolution of adaptive traits by means of natural selection in sexual species difficult or even impossible.

The relative importance of these three obstacles to the evolution of adaptive traits in response to selection is not clear. Many evolutionists suggest that epistatic interactions are the most important problem of adaptive evolution. Several distinct hypotheses suggest that the conversion of nonadditive to additive genetic variability, for example due to a radical reduction of population size, could result in an increased capacity of a population to respond to selection (Table 1); for a current review see[27] as well as comprehensive Introduction and Discussion of van Heerwaarden et al[28]. Testing such hypotheses is rather difficult. It is possible to measure the main effects of individual loci, which are responsible for the additive part of genetic variability (narrow sense heritability, h^2^); however, large experimental populations are necessary for obtaining reasonably narrow confidence intervals for the estimation of h^2^. In fact, 95% confidence intervals for the estimation of h^2 ^in most published studies include zero[29]. To measure the effects of epistatic interactions and to estimate their contribution to variability in phenotypic traits, is a much more difficult task as an unrealistically large experimental set is necessary to prove a statistically significant interaction of just two genes[30]. Statistical proof of the existence of an interaction of three or more genes is most probably beyond the capability of any experimenter. Moreover, in nearly all studies, only the epistatic effects of genes with previously proven and significant main effects were studied; consequently, the number of existing interactions must have been underestimated[31]. Because of these technical reasons, the relative importance of main effects and epistatic interactions in genetic architecture is a traditional subject of theoretical constructions[32] rather than of experimental tests[33].

**Table 1:** Differences between various theories and models related to Frozen plasticity theory.

Theory and its author	The aim	Suggested mechanism
Shifting balance theory Wright S. 1932	to explain the ability of species with large subdivided populations cross valleys in adaptive landscape	1. fragmentation of population to small subpopulations where an efficiency of selection is low 2. spreading and fixation of a new allele (that is detrimental when rare) in a subpopulation by drift 3. "Infection" of other subpopulations with individuals with new genotype originated from a successful population and the origination of new populations by these individuals

Genetic revolution Mayr E. 1954	to explain the role of founder events in speciation	1. change of balanced frequency of alleles in a split-off subpopulation due to sampling effect 2. selection for alleles with best effect on fitness instead of best-cooperator alleles

Founder-flush model Carson H.L. 1968	to explain the role of founder events in speciation	1. sampling effect due to rapid one-step reduction of a population size, 2. expansion of the population in an open uninhibited ecological niche, which relaxes all forms of selection allowing for surviving recombinants and mutants with suboptimal phenotypes (crossing valleys in the adaptive landscape) 3. reaching (or overshooting) the carrying capacity of a locality and the restoration of selection

Genetic transilience model Templeton A.R. 1980	to explain the role of founder events in speciation	1. sampling effect due to rapid one-step reduction of a population or to hybridization, 2. an increase of the amount of selectable genetic variability due to transformation of nonadditive (and therefore nonselectable) genetic variability to additive genetic variability and by higher survival probability for carriers of new mutations in the expanding population, which increases responsiveness of the population to selection 3. restoration of the population size and selection

Punctuated equilibrium Eldredge 1971	to explain the discontinuous nature of evolution and coincidence of anagenetic and cladogenetic events	various mechanisms suggested by Eldredge and Gold, including peripatric speciation and strong selection in unusual conditions on the periphery of the species' range, peripatric speciation accompanied by genetic revolution, sorting (without speciation, any evolutionary novelty is reversible due to gene flow), etc.

Frozen plasticity theory Flegr 1998	to explain why old species are microevolutionarily elastic and macroevolutionarily frozen, how frozen species can turn plastic, and the continuously decreasing rate of macroevolution	1. most polymorphism existing in an old species is sustained in it's gene pool by frequency dependent selection creating interconnected network resistant to changes of allele frequencies 2. most new (potentially useful) alleles are captured in this elastic network of alleles due to pleiotropy and its effect on (stabilized) frequencies of old alleles 3. in small splitted-off populations balancing on the edge of extinction for several generations, a decrease in strength of selection, including frequency dependent selection, will occur, and most genetic polymorphism will disappear due to drift 4. after expansion of population size, now large genetically uniform population turns evolutionary plastic - new advantageous mutations can spread in the network-free population by selection 5. traits resistant to thawing accumulate in the gene pool by sorting on the basis of stability

In fact, the Punctuated equilibrium theory in its current form was published in 1972 by Eldredge and Gould and the Frozen plasticity theory in 2008 by Flegr.

Based on the results of selection experiments, it can be argued that frequency-dependent selection rather than epistasis is the most serious barrier to adaptive evolution either by means of natural selection or by interallelic competition in sexual organisms (and possibly in asexual organism as well, see below). In most experiments, the selected traits with starting intrapopulation variability respond to selection pressure and start changing in the early stage of the experiment. However with increasing generations, or with increasing departure of the trait from its original value, the response to selection decreases and at some point, i.e. at the selection plateau, the trait stops responding at all[18]. When the selection stops, the trait returns to its original form during several generations (which, by the way, shows that the genetic variability for the given trait is still present and that the evolutionary response to selection was not stopped by an exhaustion of genetic variability). The genetic homeostasis causing the return to the original form is caused by decreased viability or fertility of individuals with the most extreme form of the selected trait. It is not the extreme form of the selected trait itself but the accompanying phenotypic changes, products of pleiotropy or compensatory changes allowing greater viability for individuals with the extreme form of the trait, what are probably responsible for decreased fitness of these individuals. For example, some breeds of dogs selected for large size suffer high rates of cancer because of the increased frequency of alleles that promote both large size and cancer[34]. When the selection for an extreme form of a trait stops, the carries of this trait are penalized by natural selection and are eliminated from the population over the course of several generations. The return to the original form (and original fitness) is not perfect in small populations because some alleles, those which became rare due to selection, are already eliminated from the gene pool of the population by drift, and cannot return to the closed population after the selection has ended. The most important message of these experiments is that the resistance of a population to selection is not present from the very beginning of an experiment but appears only after the frequency of alleles in the population has been changed by the selection. If epistasis were the major obstacle to adaptive evolution, the selection would be ineffective from the very beginning of the experiment. If the major obstacle were the dependence of a trait's influence on fitness on the presence of a particular combination of other traits in an individual, then such effect could not be observed in artificial selection experiments. Here, the experimenter, instead of the fertility or viability of an individual, decides what influence a particular trait will have on the reproduction success of an individual. Most probably, epistasis and context dependence of the influence of a trait on fitness, which critics of the Darwinian/Fisherian model of evolution (including Dawkins - compare his rowing team analogy argument presented in Selfish Gene) consider to be the main constraints to evolutionary response to selection, just slow down the adaptive evolution in sexual organisms. Frequency-dependent selection, the role of which has been highly neglected in this context, is most probably responsible for the final loss of the capacity of a population and a species to respond to artificial and natural selection. The major role of epistasis, together with pleiotropy, is that they interconnect the fates of different alleles of various loci in a single elastic network. If each gene coded just a single trait and each trait were coded by just one gene, the phenomenon of frequency dependence of the influence of the trait on fitness would concern a relatively low number of traits. These traits would be kept stable in the population; however, other traits would easily respond to selection. In real organisms, wherein the influences of particular genes are interconnected with those of many other genes in all regions of the genome, the phenomenon of frequency dependence concerns much more, possibly even all, genes. For example, an increase in body size could increase the chances of an individual in intrasexual combat and could therefore be the subject of sexual selection. In large males, the alleles with positive influence on heat removal are automatically advantaged and their frequency increases in the population. Let us imagine that some of these alleles have lethal or sublethal influence on their carrier in the homozygotic state. With an increasing mean body size of males in the population, the frequency of the allele for efficient heat removal increases. The frequency of subvital homozygotes with two copies of the allele increases with the square of the frequency of the allele; if an interaction of alleles of several loci were in play, the steepness of decrease of average fitness of the organisms in the population could be even higher, possibly stepwise. This or similar phenomenon could slow down and finally stop the response of a population to selection of any intensity.

The hypothesis known as the streetcar evolution model[35,36] suggests that this kind of genetic constraints can only temporarily stop the evolution of a population or a species; the phenotypically maladaptive equilibriums are just transient stops of the "streetcar". Sooner or later, a new mutation enables the population to circumvent a particular constraint, and adaptive evolution resumes. In the previous hypothetical example, a new allele could arise by mutation capable of increased heat removal without negative effect on the viability of the organism. The problem is that "blind and opportunistic" evolution cannot stop increasing the frequency of other heat removal-associated alleles when a new allele without any side effects appears. The new side-effect-free allele helps the population increase body size in response to sexual selection, i.e. to reach a further streetcar stop; still the changed average body size results in an increased frequency of side-effect-associated alleles and therefore decreased average viability of the population. More importantly, the rate of accumulation of constraint-building alleles could be higher than that of the accumulation of constraint-removing alleles in a polymorphic population. It is not clear which rate is higher in real systems; however, the paleontological records vital to 30 years worth of discussions of the punctuated equilibrium model of evolution suggest that, without a speciation event, the streetcar usually stays in the stop permanently[19].

As has already been mentioned, the phenomenon of frequency-dependent selection plays a very important role in sexual organisms where in every generation the alleles of two parents meet each other in new zygotes. The selective value of an allele depends, for example, on the probability that the same allele is present in chromosomes of both paternal and maternal origin, which depends on the frequency of the allele in the population. This source of frequency dependence of the selective value of alleles does not exist in asexual organisms; however, some sources of frequency dependence are present even here. For example, the hawk and dove model discussed above, is in principle a model of interaction of two asexual species or strains. Frequency dependence could take part in ecological interactions between host and parasite. The parasite is often specialized for exploitation of the most common form of its host[37], for example a host with the most common MHC alleles. Similarly, the immune system of a host is usually adapted to the most common strain of the parasite. An asexual species can be a mixture of phenotypically and genetically different strains with differing strategies to exploit resources from their environment. The frequencies of particular strains could change in response to changes in the environment (to changes in the intensity of shooting hawks); they will probably express a tendency to return to some equilibrium state. The long-term competition of strains (strategies) with selective values dependent on their frequency in the population could be an important source of species cohesion even in asexual species.

## Results

### Mechanism of Adaptive Evolution in Sexual Organisms

**Figure 2:**
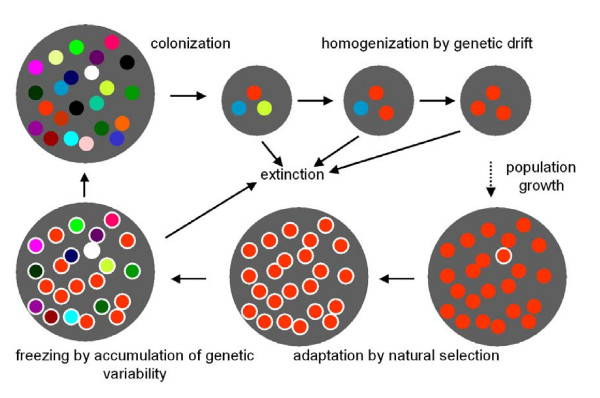
**Role of peripatric speciation in adaptive evolution of sexually reproducing organisms**. It must be emphasized that extinction is a more probable fate for a small population than expansion. However, unsuccessful speciation events are not interesting from the perspective of evolution.

It was argued above that adaptive evolution by means of Darwinian selection of individuals in a population, as well as by means of Dawkinsian competition between alleles in individual loci, is very difficult, if not impossible, in populations of sexual organisms. It is evident, however, that adaptive evolution operates on Earth and that this evolution is especially and extremely efficient in sexual organisms. It follows that a situation must occur, in which all three barriers to adaptive evolution are lifted, or at least temporarily operate with decreased efficiency. It is highly probable that peripatric speciation plays the key role in adaptive evolution in sexual organisms[38]. In this form of allopatric speciation, a small population splits from a large one via an invasion of a small number of individuals into an isolated locality beyond the geographic range of the mother species (Fig. 2). Removed from any genetic and ecological contact with the mother species, this split-off population subsequently evolves into a new species with a different phenotype. During the isolation, many alleles incompatible with those in the mother population accumulate in the new species[39,40], which creates a sufficiently strong reproductive barrier. With such barriers intact, the new species could subsequently expand into the area of the mother species. When the ecology of the new and old species differs, both species can coexist in the same area, otherwise either the new species or the old one is eliminated. From the point of view of the capacity for adaptive evolution, the most important fact is that the founders of a new species only bring a small part of genetic variability from the original to the new population. It is evident that common alleles, which are usually sustained in the population by some form of frequency-dependent selection, are preferentially transferred to the new population. During a population bottleneck, i.e. in the first stage of peripatric speciation, the population rids itself of most of the originally present rare alleles; however, it retains most of the common alleles that are responsible for genetic homeostasis [41-44]. In the second stage of peripatric speciation, the new population of organisms (still adapted to the conditions of their original geographic range) survives in the different conditions of their new area. Expectedly, the size of such a population remains low for many generations, and extinction is their most probable destiny, either because of bad luck or inbreeding depression (or both). In small populations, chance rules the destiny of particular organisms as well as particular alleles, rather than any form of selection. (No matter whether you are a dove or a hawk, your biological success is just a question of chance.) Therefore, in the second stage of peripatric speciation, the genetic variability that had been sustained by frequency-dependent selection in the original large population disappears due to drift. If the colonization is successful, the population must, at some point, expand. (Unsuccessful colonizations are not interesting from the perspective of evolution, and evolution has time enough to repeat each unsuccessful experiment thousands of times.) At that very moment, conditions optimal for adaptations to evolve by means of selection are present. The population is large; therefore selection rather than chance rules the destiny of an individual. Still, the population is genetically uniform; thus a new mutation meets the same combination of alleles in each generation and has the same influence on the phenotype of the organism and its fitness. The alleles sustained in the population by means of frequency-dependent selection are either not present, or rather rare in the gene pool of the population. In such a population, both Darwinian selection of individuals for best fitness and Dawkinsian selection of most efficiently replicating alleles could work to produce new adaptive traits. Through time, more and more mutations with frequency-dependent influence on fitness are captured in the population. And hence, the genetic variability of the population increases and an originally plastic species builds up an interconnected network of cooperating and competing strategies, thereby becoming elastic.

The return to the elastic state is probably not a yes/no process. Some properties of organisms develop elasticity rather quickly; other properties (different in different taxa) could stay plastic for longer time. However, the loss of plasticity is probably an autocatalytic process. As the amount of genetic polymorphism (as well as the number of already frozen properties) increases, the probability that the biological value of a new mutation will be dependent on its own frequency and will therefore be captured in an interconnected network of polymorphic genes, will also increase. Therefore, the conversion of a species from plastic to elastic states could in fact be rather abrupt, and the difference between plastic and elastic species could in fact be rather sharp. Before such conversion, a species can respond to environmental change by radical change in its phenotype. In the frozen stage, a species is not able to respond to the same environmental change through a corresponding change of its phenotype[45].

This model of adaptive evolution, called the theory of frozen plasticity[14,46], is, in a certain sense, a return from the Dawkinsian to the original Darwinian model of evolution. The main difference is that according to this new theory, all of the processes described by Darwin and Dawkins do operate just after the birth of a species by peripatric speciation - while it is still plastic. However most of the time, i.e. 98-99% of a species duration (as estimated by Gould on the basis of paleontological data[47]), sexual species are evolutionarily frozen and can only passively wait for such changes in their environment that cause either their extinction, or for the highly improbable event of a return of a part of the population to the plastic state due to peripatric speciation.

### Decreasing rate of macroevolution

Paleontological data suggest that the rate of evolution and amount of interspecies and intraspecies variability decreases with the age of the phylogenetic line. Several independent studies have shown that maximum biodiversity, and disparity of a clade in particular, is achieved rather early after the origin of the phylogenetic line (clade) [22,48,49]. For example, Webster [50] has reported that the frequency and extent of morphological variation in 982 trilobite species are greatest early in the evolution of the group. He has shown that "the proportion of species with at least one polymorphism drops sharply between the Middle Cambrian (75%) and Late Cambrian (8%), then rises to 40% in the Early Ordovician (coincident with the first sampling of the diverse phacopid and proetid orders), after which there is a progressive decline through the Middle Devonian (1%), interrupted only by a particularly low value (0%) in the Late Silurian. No polymorphism was recorded in character-state coding among the 23 post-Devonian species. Genera originating in the Cambrian had shorter average durations than genera originating in the post-Cambrian [51], resulting in accrual of lower species-level diversity per genus."

These phenomena, which have no support in current evolutionary theory, could have a common cause, namely the continuous irreversible freezing of more and more traits during the evolution of a clade. It is sure that traits differ in resistance to transition from frozen to plastic in response to a reduction of genetic polymorphism. For some traits, this process is likely to happen readily and can be achieved by a relatively small reduction in genetic polymorphism. For other traits, the transition from frozen to plastic is difficult or even impossible, as it needs an unrealistically small founding population and an unrealistically long period of persistence of such a small population in an extinction-prone state. On a macroevolutionary time scale, more and more traits pass into the permanently frozen state due to a universal process of sorting on the basis of stability [47]. The stable traits (and systems and anything) persist while the unstable traits (and systems and anything) pass away. An example of a stable trait is a trait that is coded by many genes that are substitutable in their effect. The mutation of an allele in one locus (or several loci) does not result in the change of such a trait. At the same time, the mutation in all loci is highly improbable especially if, due to pleiotropy, the genes in particular loci also influence other traits. Another source of the evolutionary stability of a trait is frequency-dependent selection, particularly the steep dependence of fitness on the frequency of a particular allele. When the fitness of an individual sharply decreases with an increased frequency of an allele (of a particular trait, strategy), even a drastic reduction in population size cannot lead to total loss of polymorphism in a particular locus and corresponding trait. Due to dominance and especially due to epistatic interactions of more than two genes, the slope of the fitness function can be very steep. In the former case, the fitness of homozygotes with genotype aa could decrease at a rate proportional to the second power of the trait frequency. In the latter case, the rate could even be proportional to a higher power of the trait frequency. This kind of trait probably survives peripatric speciation in a polymorphic state, or polymorphism in such a trait is restored very quickly in the originating new species due to mutations.

In a new clade, a high proportion of species contain many traits that could melt during standard peripatric speciation or that are relatively plastic even on the level of a species (or even of a local population). Through time, more and more traits in more and more species turn to a semipermanently or even permanently frozen state. The representatives of a particular clade are not only less and less variable (more and more elastic - resistant to selection pressure) but also exhibit elasticity that is less and less affected by peripatric speciation. Originally, many representatives of a clade had the capacity to evolve new body plans after peripatric speciation. In the end, only some species retain this capacity and even in these species some traits had a highly limited capacity to respond to selection after peripatric speciation.

## Conclusions

The frozen plasticity theory[14,46] is a complex theory, based on many particular hypotheses concerning the mechanisms of evolutionary stasis and evolutionary change - most of which were suggested in some form by different students of evolution during the 20^th ^century. It is possible that some of these mechanisms may be wrong, or at least may not play as important a role in the discussed phenomena as the frozen plasticity theory would suggest. The existence of the plastic and frozen phases in the life of a species resulting in a punctuated equilibrium pattern of evolution[52,53] is now a widely accepted (most probably prevailing) model of the evolution of multicellular life on Earth. The frozen plasticity theory suggests that certain rather probable hypothesis on the nature of evolutionary stasis (frequency-dependent selection and pleiotropy-based elasticity of genetically polymorphic species) and evolutionary plasticity (loss of genetic polymorphism due to the founder effect during peripatric speciation, and drift following it) could have a very important impact, not only on macroevolutionary but also microevolutionary and ecological processes[14,46].

It must be emphasized that the frozen plasticity theory has a much broader spectrum of evolutionary and ecological implications than has been suggested and discussed by students of particular hypotheses. In fact, the picture of evolutionary and ecological processes presented by the frozen plasticity theory differs in many respects from that provided by the current textbook theory of evolution; see Table 2, [54] and (Flegr J.: Microevolutionary a macroevolutionary implication of Frozen plasticity theory of adaptive evolution, submitted). Most of these predictions could be tested empirically and should be analyzed in greater depth theoretically. In my (very subjective) opinion, the frozen plasticity theory, which includes the Darwinian model of evolution as a special case - the evolution of species in a plastic state, not only offers plenty of new predictions to be tested, but also provides explanations for a much broader spectrum of known biological phenomena than classic evolutionary theories.

**Table 2:** Differences between predictions of the classical theories of evolution and frozen plasticity theory of evolution.

	clasical theory	frozen plasticity theory
anagenesis and cladogenesis **^1, 2^	are independent	are coupled

divergence of species^1^	does not correlate with taxon richness	correlates with taxon richness

genetic polymorphism **^3^	accelerates evolution	decelerates evolution

species respond to selection *^4^	plastically (as plasticine)	elastically (as ruber)

species are adapted to *^5^	current environment	original environment

local and global abundance **^6^	correlate for any species	do not correlate for old species

abundance of species	is independent of species age	decreases with species age

ability of species to respond to environmental changes **^7^	is independent of species age	decreases with species age

species on islands are derived *^1^	as much as those on continents	more than those on continents

asexual species are*^8^	less adapted to their environment	more adapted to their environment

cross-pollinating species *^9^	as stable as self-pollinating species	more stable than self-pollinating species

invasive species **^10^	express average heritability	express higher heritability

domesticated species	express average heritability	express higher heritability

domesticated species	express average age	are evolutionarily younger

successful selection*^11^	has no influence on fitness	decreases fitness

rate of anagenesis in a clade*	is (on average) constant	usually decreases

two species in the same niche*	usually cannot coexist	frequently can coexist

slow long-term trends*	are hardly possible	are quite possible

Two asterisks denote the predictions that have already been tested and support the frozen plasticity model. One asterisk denotes the predictions that have not been intentionally tested but are supported by published data. ^1^[57], ^2^[58], ^3^[59,60], ^4^[17], ^5^[61], ^6^[62], ^7^[45], ^8^[63,64], ^9^[65], ^10^[66-68], ^11^[69,70]. For explanation see [46] and (Flegr J.: Microevolutionary a macroevolutionary implication of Frozen plasticity theory of adaptive evolution, submitted).

## Competing interests

The author declares that he has no competing interests.

## Reviewers' comments

### Reviewer's report 1

Rob Knight, University of Colorado, Boulder, United States

The problem of where adaptation occurs has attracted much interest over the decades, dating back at least as far as the debates between Fisher, who envisioned a single panmictic population evolving towards an optimal 'peak' in a static genetic landscape, and Wright, who envisioned an ever-shifting alliance of small populations where changes in genetic backgrounds allowed new combinations of alleles to survive and, occasionally, to flourish and sweep over the population. In this manuscript, the author provides an outline of organism- and gene-level selection processes, focusing on the observation that laboratory populations tend to revert to wild type once selection pressure is relaxed (the idea of an "elastic" population that snaps back to its initial state). The argument is that populations spend most of their time in this elastic state, and it is only under unusual circumstances, such as very small populations where many alleles are lost, that this elasticity disappears and rapid change is possible. The main supporting lines of evidence are that frequency-dependent selection (e.g. an allele conferring resistance against a specific parasite might be beneficial only when rare as when common the parasite might evolve to counter it), epistasis (i.e. whether the gene is expressed might depend on the states of other genes), and phenotype-dependent fitness effects (e.g. a gene implicated in production of the same phenotype, such as a luxuriant mustache, might contribute to differential reproductive success in males vs. females, or in 1910 vs. 2010), and therefore the same allele cannot be said to have the same consistent effect (although it should be noted that this issue has been examined fairly thoroughly in the literature, and the usual response, used e.g. by Dawkins, is that the models are a useful guide when average effect sizes are used -- however, this view has been criticized and counterexamples can be constructed where average fitness effects give misleading results). The author argues that, of these effects, frequency-dependent selection is most important, and draws on Mayr's theory of peripatric speciation to argue that small peripheral populations are able to escape frequency-dependent selection, find new optima, and outcompete the rest of the population with rapid adaptive change.

The main issues I see with the manuscript are (i) the need to distinguish the "frozen plasticity" theory carefully from Wright's shifting balance model, and in particular to ascertain whether the evidence for and against that model also applies to this model, and (ii) the great benefits that could be introduced through a quantitative modeling approach rather than a qualitative discussion based on general principles. It might also be interesting to include a discussion of recent evidence from Peter and Rosemary Grant and others that strong directional selection operates in opposite directions from year to year, and thus what we see in natural populations may be average responses over a longer timescale. It could reasonably be argued that quantitative modeling is outside the scope of the present work and that not every evolutionary mechanism need be taken into account, but the omission of Wright from both the present manuscript and the accompanying one provided for the referees' information really needs to be corrected.

A few additional comments:

- The history of and relationship between organism-level and gene-level selection will likely be familiar to many readers and can be condensed substantially (while leading to the same italicized punchline).

- Dawkins explicitly addresses the issue of the same allele having different effects in different bodies with the rowing team analogy in chapter 5 of The Selfish Gene: although different individuals will perform better or worse in different crews, individuals who on average find themselves in fast boats are more successful, and this is due to intrinsic differences in ability (in the context of the kinds of crews one is likely to find). Although this analogy has been criticized by some authors as overly simplistic, the point that what matters are long-run averages rather than individual outcomes is important.

- Frequency-dependent selection on genes has received a great deal of attention in the literature and the reader might be misled by the discussion on p 6 that the concept is being introduced for the first time in the present work -- it would be useful to delineate more clearly what the present work is adding to existing treatments.

- Similarly, the effects of pleiotropy and epistasis have received extensive treatments from modelers that it would be useful to mention in the discussion on pp. 7-8. In particular, the effects of frequency-dependent selection can be modeled directly using simple population genetics simulations. The concern about the cited lab experiments is that the reversion may or may not be due to frequency-dependent selection: in some cases, we may be seeing loss of alleles that are deleterious under most conditions but not under the artificial selection conditions (e.g. genes for stress tolerance that slow growth under normal conditions, of which there are many), in some cases we may be seeing multiple mutually exclusive combinations of alleles that "solve" the same problem, which recombine to give more typical phenotypes, etc. In general, the ideas in this manuscript would benefit from a mathematical or simulation treatment, which would allow direct evaluation of the conditions under which central or peripheral populations would be likely to adapt the fastest.

- The discussion about how "there is no such thing as an allele, or a mutation, for large body size" would benefit greatly from a discussion of some of the literature on "genes for" in the philosophy of biology over the past 20 years, especially the work of Susan Oyama, Paul Griffiths, Eva Neumann-Held, Karola Stotz, Kim Sterelny, and others. Considerable conceptual work has been done on what biologists mean when they talk about a "gene for" something, especially in the context of debates about genes as context-sensitive difference makers and genes as developmental resources. Fred Nijhout's pioneering work on biochemical models of butterfly eyespots is especially relevant here. See also Brandon & Nijhout's more recent work on gene vs. genotype level selection, e.g. [55].

- The description of plastic evolution on p 14 reads as very similar to Wright's shifting balance model and elaborations on this model by later population geneticists. It would be useful for this work to more clearly delineate the similarities and differences between the two theories. For a good review of Wrightian vs. Fisherian evolution see [56].

### Author's response

I would like to thank to Dr. Knight for very nice introductory paragraph of his review, which, in fact, can serve a second, pregnant summary of my paper. In accord with his suggestions, I have made more explicit what conceptions are new and what old. The mechanism responsible for evolutionary elasticity of frozen sexual species, the most important part of the frozen plasticity theory, represents a new combination of mostly very old mechanisms. Possibly, the most important new idea is that of the ubiquity of frequency dependent selection in sexual species due to omnipresent encounters and conflicts of paternal and maternal copies of genes in zygotes in each generation (the analogy of hawks and doves). Therefore, the mechanism of evolutionary elasticity suggested by frozen plasticity theory is first of all the result of a synthesis of Ernest Mayr's genetic revolution model with John Maynard Smith's theory of evolutionarily stable strategies. The mechanism of transfer of a species from elastic to plastic state is most related to that postulated by Sewall Wright as the first stage of his shifting balance model of adaptive evolution in subdivided populations. In his model, the small size of local populations enables new genotypes to cross valleys in the adaptive landscape due to the released strength of negative selection against suboptimal phenotypes. In my model, the small size of split off populations balancing on the edge of extinction releases the strength of frequency dependent selection, which enables genetic drift to prune away most of the polymorphism responsible for evolutionary elasticity within a species. The search for a mechanism of decreasing rate of macroevolution and decreasing variability of species in macroevolutionary time-scale, i.e. the third part of frozen plasticity theory, was inspired by the existence of phenomena described by Stephen J. Gould, Mark Webster and John A. Davison. And last, but definitely not least, I must acknowledge that my whole search for a new theory of adaptive evolution in sexual species was mainly inspired by reading the ingenious critique of the old Fisherian theory in Richard Dawkins' Selfish Gene.

I added a new Table 1, which summarizes differences between frozen plasticity theory and several related models, including Wright's shifting balance model. I believe that this is the most important (and most useful) change performed in the corrected version of the manuscript.

I decided not to discuss present conceptions of a gene (but I added recent references) because it can divert the attention of readers from the main subject of the paper. By my opinion, the most important mechanism responsible for evolutionary elasticity of sexual species is a frequency dependent selection. I believe that the evolutionary plasticity of sexual species would disappear even in systems with frequency dependent selection and without any epistasis (but probably not in systems without pleiotropy).

### Reviewer's report 2

Fyodor Kondrashov, Centre for Genomic Regulation, Barcelona, Spain

Throughout reading this manuscript I found myself disagreeing with the author on several different levels. First and foremost, I disagree with the main premise of this manuscript. Second, the manuscript is fraught with misleading statements pertaining to the current state of population genetics and evolutionary theory, omissions of previous theoretical work and inaccurate usage of uncommon terminology. Finally, while this paper from the title appears to be about theory, not a single definition or equation is provided that could have somehow helped my understanding of the essence of the "frozen plasticity theory" that the author wanted co convey to the reader.

The manuscript starts with several verbal arguments that lead the author to conclude that "adaptive evolution by means of Darwinian selection ... is very difficult, if not impossible, in populations of sexual organisms." I could not disagree more, and the literature that, in my opinion, demonstrates the action of positive selection in sexual organisms is too large to make a meaningful review here, and includes both theoretical treatments of this subject (such as the advantage of sex due to recombining of beneficial alleles or the theoretical work on sympatric speciation) and numerous papers reporting the action of positive selection in natural populations. The author presents four verbal arguments that in his opinion rejects the possibility of Darwinian evolution in natural populations. 1) frequency-dependent selection 2) epistatic interactions 3) genotype-environment interactions and 4), which the authors does not articulate directly, but it seems that he believes that the stochastic nature of recombination implies that "the unique combination of genes of an excellent individual will be diluted and its relatives will in no way differ from the relatives of any other individual" and that "in sexually reproducing organisms neither genotype nor phenotype is inherited from parents to offspring".

To formally refute all four statements would require several courses in Biology. Briefly, neither of the four issues raised present an issue for possibility of adaptive evolution in sexually reproducing organisms. 1) It is unlikely that every trait, gene or DNA site is subject to very strong frequency-dependent selection and, therefore, the first barrier is unlikely to be substantial in nature. 2) Although strong epistasis can limit the rate of adaptive evolution more profoundly in sexual than asexual populations, any epistatic fitness ridge that prohibits evolution completely in a sexual population would also do so in an asexual population. 3) Genotype-environment (which the author calls genotype-phenotype) interactions are also not a complete barrier to adaptive evolution as long as an allele has any measurable level of heritability and is not completely overwhelmed by a changing environment. 4) Finally, with regard to transmission of alleles between generations in a sexual population it should be recognized that while the offspring get an intermediate genotype of the parents this in no way prohibits positive selection for a specific allele and does not imply that "genotype is not inherited".

In addition to these general misconceptions the manuscript is also littered with wrong and otherwise problematic statements some of which are neither right or wrong but just inappropriate.

Thus, while I am in agreement that frequency-dependent selection, epsitatic interactions and environmental effects pose interesting evolutionary questions, the authors does not do justice to these issues in modern evolutionary biology in the current manuscript. I cannot suggest specific ways to improve this manuscript as I believe it to be beyond salvation.

### Author's response

In the paper, I try to convince readers that (due to the effect of pleiotropy) sexual reproduction, namely processes of segregation and recombination of genetic material, makes frequency dependent selection so ubiquitous that Fisherian evolution of adaptive traits by individual selection (or Dawkinsian evolution by intralocus competition of alleles) is practically impossible in a normal genetically polymorphic population. Evidently, I did not succeeded in convincing Fyodor Kondrashov. It would be rather unproductive to try to repeat or elaborate my arguments here in more details. If the reader (or the referee) would like to hear the arguments in a less condensed form, he or she can read the book Frozen Evolution.

I would just like to correct here one misunderstanding of Dr. F. Kondrashov. The third barrier to adaptive evolution in sexual organisms is neither genotype-phenotype interaction nor genotype-environment interaction but the trait-trait interaction, the fact is that "the effect of the same trait on the fitness of an individual is usually dependent on combination of other traits carried by the same individual". The trait (e.g. aggression) that increases fitness of an individual carrying certain combination of traits (e.g. the physically strong individual) can decrease fitness of another individual carrying another combination of traits (e.g. in weak individuals). Due to such trait-trait interaction the spreading of many (large majority?) of traits in sexual species should be studied using game theory, rather than classical population genetics theories.

Fyodor Kondrashov misses equations in the paper describing my theory. By my opinion, however, equations are very useful for describing or even refuting particular models (see K.R. Popper), however, they are much less useful for describing and more or less useless for refuting complex theories (see T.S. Kuhn). The frozen plasticity theory of adaptive evolution in sexual organisms aims to substitute the previous selfish gene theory, which itself substituted the previous theory of individual selection thirty years ago. Let us recall that neither Selfish Gene, nor Origin of Species contains any equations.

Finally, I would like to thank Dr. Fyodor Kondrashov, who is not only a referee but also a coeditor of this paper for Biology Direct, for his fairness in handling a manuscript with which he does not agree, and for his courage to write an open review for a work most probably strange - but with a certain non-negligible potentiality of importance. I suppose that most colleagues in his position would somehow flinch from writing the review.

### Reviewer's report 3

Massimo Di Giulio CNR, Lab Mol Evolut, Inst Genet & Biophys, Naples, Italy (nominated by David H. Ardell, University of California, Merced, California, United States)

The paper "Elastic, not plastic species: Frozen plasticity theory and the origin of adaptive evolution in sexually reproducing organisms" presents and discusses the author's theory of frozen plasticity that should provide an alternative explanation of the evolution of adaptive traits in sexual organisms. After reading its extended, elaborated discussion, I am more or less convinced that the theory is original and correct; however, I am not convinced that the correct parts of the theory are new and that the new parts are correct.

The suggested mechanisms of the transition between the elastic and plastic states of a species remind me of Ernest Mayr's theory of genetic revolution. The author should clearly state what the differences are between his theory and fifty-year-old Mayr's model.

The author supports his theory with the results of selection experiments showing that the evolution of populations by directional selection ends by reaching a selection plateau. It is not clear, however, whether this is a regular or rather exceptional result of such experiments. The efficiency of practical breeding programs suggests that the populations of the bred animals and plants evolve towards higher productivity even after tens of years of artificial selection.

The author claims that the punctuated pattern of evolution is now a widely accepted model of the evolution of multicellular life on the Earth. However, it is not clear what he means by "widely accepted". Does he mean " the prevailing model" or just "a widespread model"?

Table 1 (which is not referred to in the manuscript and this should be corrected) says that the aim of the frozen plasticity theory is to explain not only why old species are elastic and how an elastic species turns plastic during peripatric speciation but also the "decreasing rate of macroevolution". However, the third mechanism is discussed in the accompanying manuscript, but is not mentioned in the reviewed manuscript. Moreover, I am not sure whether the phenomenon of the decreasing rate of macroevolution really exists (at least I do not think that it is widely accepted in the standard evolutionary theories) and therefore whether the explanation for this phenomenon is really needed.

In the Summary and Conclusions, the author says that the evolutionary and ecological implications of the theory are discussed in the accompanying manuscript. However, this aforementioned manuscript (available to me as a referee) is not to be published in the Biology Direct. Therefore, it should not be referred to as the accompanying manuscript in the reviewed paper. Generally, the reviewed paper should be a stand-alone paper. The differences in predictions between the classical evolutionary theories of adaptive evolution and frozen plasticity theory of adaptive evolution should be briefly recapitulated.

The results of famous Van Valen's Red Queen hypothesis have shown that species do not age (the probability of extinction of a species during a certain constant time interval does not correlate with length of its existence). However, according to the frozen plasticity theory the old frozen species should have higher probability of extinction than young plastic species. How to explain this contradiction?

In conclusion, I am convinced that the author is absolutely right when saying that many of the mechanisms postulated by the frozen plasticity theory as playing a key role in the evolution "may be wrong or at lest may not play as important a role in the discussed phenomena as the frozen plasticity theory suggests". Still, I also believe that this paper is worthy of publishing, as it may inspire biologists and paleontologists to study systematically some potentially important but rather neglected phenomena.

### Author's response

The differences between frozen plasticity theory and related models are listed anew in Table 1. I agree with Dr. Di Giulio - Mayr's genetic revolution model most closely resembles the mechanism of the transition of a species from elastic to plastic state suggested by frozen plasticity theory. I believe that if Mayr would know the theory of evolutionarily stable strategies and the paleontological evidence for a punctuated character of evolution in the 50ies, he would probably have suggested the frozen plasticity theory many decades before me. His model of the transition of species to a plastic state is based on the drastic change of frequency of alleles due to sampling effect during a bottleneck event. My model of transition is based on elimination of genetic polymorphism (stabilized in large populations by frequency dependent selection) by genetic drift during generations after the bottleneck event, the mechanism similar but not identical with that operating during the first stage of Sewall Wright's shifting balance model.

Surprisingly, results of laboratory selection experiments performed on sexual organisms are rather sparse. Therefore, I am not able to say how common the phenomenon of the genetic homeostasis is. The usual targets of breeding programs are domesticated species that have been exposed to drastic bottleneck effect during domestication (probably even before domestication, see [46]) and can therefore be evolutionarily plastic.

I explicitly state that the punctuated equilibrium model is probably the prevailing model of evolution of multicellular species in the corrected version of the manuscript. I hope that paleontologists would appreciate that the frozen plasticity theory provides a theoretical background for their current prevailing model of evolution.

I would like to thank to Dr. Di Giulio for reminding me of the omission of the third aim of frozen plasticity theory, namely explaining the decreasing rate of macroevolution. A new chapter has been added to the corrected version of the manuscript and several references supporting the existence of this important but so far unexplained phenomenon have been added.

Table 2 lists important evolutionary and ecological implications of frozen plasticity theory; also, references to papers dealing with this subject have been added to the corrected version of the paper.

I believe that there is no contradiction between frozen plasticity theory and Van Valen's data. The famous Van Valen study was focused on probabilities of extinctions of mammal genera, not mammal species. Moreover, there was only a small probability that young and therefore still plastic species, which represent about 1-2% of all species in any time, was included in original set of studied species. Of course, even among the frozen species, the older species are probably more obsolete then younger species and therefore have a higher probability of extinction. However, in randomly fluctuating environmental conditions the correlation between the age of frozen species and their probability of extinction is probably rather loose.
